# Supplementary material for: Unravelling the genetic diversity of water yam (Dioscorea alata L.) accessions from Tanzania using simple sequence repeat (SSR) markers
Source: PLoS One. 2023 May 31;18(5):e0286480. doi: 10.1371/journal.pone.0286480 (PMC10231824; doi:10.1371/journal.pone.0286480)

PLOS ONE

Unravelling the genetic diversity of water yam (Dioscorea alata L.) accessions from Tanzania using simple sequence repeat (SSR) markers

--Manuscript Draft--

**Manuscript Number:**	PONE-D-22-32759
**Article Type:**	Research Article
**Full Title:**	Unravelling the genetic diversity of water yam (Dioscorea alata L.) accessions from Tanzania using simple sequence repeat (SSR) markers
**Short Title:**	Genetic diversity and <i>Dioscorea alata</i> L.
**Corresponding Author:**	Joseph Massawe<br>The University of Dodoma<br>Dodoma, Dodoma TANZANIA, UNITED REPUBLIC OF
**Keywords:**	<i>Dioscorea alata</i>; Genetic diversity; Greater yam; SSR marker
**Abstract:**	Water yam (Dioscorea alata L.) is among the most cultivated species used as a source of food and income for small-scale farmers in Tanzania. However, little is documented about Dioscorea species available in Tanzania, including their genetic diversity. This study used ten polymorphic microsatellite markers to determine the genetic diversity and relationship of 63 D. alata accessions from six major producing regions. Results revealed a polymorphic information content (PIC) of 0.63, while the number of alleles per locus ranged from 4 to 12 with a mean of 7.60. The expected heterozygosity ranged from 0.17 to 0.74, with a mean of 0.49, which suggests moderate genetic diversity of D. alata accessions. Kagera region had the highest mean number of (1.5) private alleles. Analysis of molecular variance revealed that 91% of the variation was attributed to within population while among population contributed 9% of the total variation. The highest Nei's genetic distance (0.65) was for accessions sampled from Arusha and Mtwara regions. Principal coordinate analysis and Unweighted Paired Group Method using Arithmetic (UPGMA) analysis grouped D. alata accessions into two major clusters regardless of geographical origin and local names. The Bayesian structure analysis confirmed the two clusters obtained in UPGMA and revealed an admixture of D. alata accessions in all six regions suggesting farmers' extensive exchange of planting materials. These results are helpful in the selection of D. alata accessions for breeding programs in Tanzania.
**Order of Authors:**	Joseph Innocent Massawe
	Gladness Elibariki Temu
**Additional Information:**	
**Question**	**Response**
**Financial Disclosure**<br><br>Enter a financial disclosure statement that describes the sources of funding for the work included in this submission. Review the submission guidelines for detailed requirements. View published research articles from *PLOS ONE* for specific examples.<br><br>This statement is required for submission and **will appear in the published article** if the submission is accepted. Please make sure it is accurate.	JIM received the research fund under the MoEST 2018 sponsorship. This study was funded by Ministry of Education, Science and Technology (Tanzania). The funder can be accessed via https://www.moe.go.tz/sw. The funders had no role in study design, data collection and analysis, decision to publish, or preparation of the manuscript.

Powered by Editorial Manager® and ProduXion Manager® from Aries Systems Corporation

**Unfunded studies**<br>Enter: *The author(s) received no specific funding for this work.*<br><br>**Funded studies**<br>Enter a statement with the following details:<br>• Initials of the authors who received each award<br>• Grant numbers awarded to each author<br>• The full name of each funder<br>• URL of each funder website<br>• Did the sponsors or funders play any role in the study design, data collection and analysis, decision to publish, or preparation of the manuscript?<br>• **NO** - Include this sentence at the end of your statement: *The funders had no role in study design, data collection and analysis, decision to publish, or preparation of the manuscript.*<br>• **YES** - Specify the role(s) played.<br><br>* typeset	
**Competing Interests**<br><br>Use the instructions below to enter a competing interest statement for this submission. On behalf of all authors, disclose any competing interests that could be perceived to bias this work—acknowledging all financial support and any other relevant financial or non-financial competing interests.<br><br>This statement is required for submission and **will appear in the published article** if the submission is accepted. Please make sure it is accurate and that any funding sources listed in your Funding Information later in the submission form are also declared in your Financial Disclosure statement.<br><br>View published research articles from *PLOS ONE* for specific examples.	The authors have declared that no competing interests exist.

**NO authors have competing interests**<br><br>Enter: *The authors have declared that no competing interests exist.*<br><br>**Authors with competing interests**<br><br>Enter competing interest details beginning with this statement:<br><br>*I have read the journal's policy and the authors of this manuscript have the following competing interests: [insert competing interests here]*<br><br>* typeset	
**Ethics Statement**<br><br>Enter an ethics statement for this submission. This statement is required if the study involved:<br><br>• Human participants<br>• Human specimens or tissue<br>• Vertebrate animals or cephalopods<br>• Vertebrate embryos or tissues<br>• Field research<br><br>Write "N/A" if the submission does not require an ethics statement.<br><br>General guidance is provided below. Consult the submission guidelines for detailed instructions. **Make sure that all information entered here is included in the Methods section of the manuscript.**	This study involved a field research whereby plant materials were collected.<br><br>The permission to collect plant materials was granted by the University of Dar es Salaam with the permit number AB3/12(B).

Powered by Editorial Manager® and ProduXion Manager® from Aries Systems Corporation

**Format for specific study types**<br><br>**Human Subject Research (involving human participants and/or tissue)**<br>• Give the name of the institutional review board or ethics committee that approved the study<br>• Include the approval number and/or a statement indicating approval of this research<br>• Indicate the form of consent obtained (written/oral) or the reason that consent was not obtained (e.g. the data were analyzed anonymously)<br><br>**Animal Research (involving vertebrate animals, embryos or tissues)**<br>• Provide the name of the Institutional Animal Care and Use Committee (IACUC) or other relevant ethics board that reviewed the study protocol, and indicate whether they approved this research or granted a formal waiver of ethical approval<br>• Include an approval number if one was obtained<br>• If the study involved *non-human primates*, add *additional details* about animal welfare and steps taken to ameliorate suffering<br>• If anesthesia, euthanasia, or any kind of animal sacrifice is part of the study, include briefly which substances and/or methods were applied<br><br>**Field Research**<br><br>Include the following details if this study involves the collection of plant, animal, or other materials from a natural setting:<br>• Field permit number<br>• Name of the institution or relevant body that granted permission	
**Data Availability**<br><br>Authors are required to make all data underlying the findings described fully available, without restriction, and from the time of publication. PLOS allows rare exceptions to address legal and ethical concerns. See the PLOS Data Policy and FAQ for detailed information.	Yes - all data are fully available without restriction

A Data Availability Statement describing where the data can be found is required at submission. Your answers to this question constitute the Data Availability Statement and **will be published in the article**, if accepted.<br><br>**Important:** Stating 'data available on request from the author' is not sufficient. If your data are only available upon request, select 'No' for the first question and explain your exceptional situation in the text box.<br><br>Do the authors confirm that all data underlying the findings described in their manuscript are fully available without restriction?	
**Describe where the data may be found in full sentences. If you are copying our sample text, replace any instances of XXX with the appropriate details.**<br><br>• If the data are **held or will be held in a public repository**, include URLs, accession numbers or DOIs. If this information will only be available after acceptance, indicate this by ticking the box below. For example: *All XXX files are available from the XXX database (accession number(s) XXX, XXX.).*<br>• If the data are all contained **within the manuscript and/or Supporting Information files**, enter the following: *All relevant data are within the manuscript and its Supporting Information files.*<br>• If neither of these applies but you are able to provide **details of access elsewhere**, with or without limitations, please do so. For example:<br><br>*Data cannot be shared publicly because of [XXX]. Data are available from the XXX Institutional Data Access / Ethics Committee (contact via XXX) for researchers who meet the criteria for access to confidential data.*<br><br>*The data underlying the results presented in the study are available from (include the name of the third party*	This information will be available after acceptance.

and contact information or URL).<br>• This text is appropriate if the data are owned by a third party and authors do not have permission to share the data.<br><br>* typeset	
Additional data availability information:	Tick here if the URLs/accession numbers/DOIs will be available only after acceptance of the manuscript for publication so that we can ensure their inclusion before publication.

Powered by Editorial Manager® and ProduXion Manager® from Aries Systems Corporation

Manuscript
Click here to access/download;Manuscript;Genetic diversity of water yam.doc

Unravelling the genetic diversity of water yam (*Dioscorea alata* L.) accessions from Tanzania using simple sequence repeat (SSR) markers

Short title: Genetic diversity and (*Dioscorea alata* L.)

Joseph I. Massawe*[1, 2#a] and Gladness E. Temu[2]

[1]Department of Molecular Biology and Biotechnology, University of Dar es Salaam, Dar es Salaam, Tanzania

[2]Department of Biology, University of Dodoma, Dodoma, Tanzania

*Corresponding author: joseph.innocent92@gmail.com (JIM)

[#a]Current Address: Department of Biology, University of Dodoma, Dodoma, Tanzania

// Abstract

Water yam (*Dioscorea alata* L.) is among the most cultivated species used as a source of food and income for small-scale farmers in Tanzania. However, little is documented about *Dioscorea* species available in Tanzania, including their genetic diversity. This study used ten polymorphic microsatellite markers to determine the genetic diversity and relationship of 63 *D. alata* accessions from six major producing regions. Results revealed a polymorphic information content (PIC) of 0.63, while the number of alleles per locus ranged from 4 to 12 with a mean of 7.60. The expected heterozygosity ranged from 0.17 to 0.74, with a mean of 0.49, which suggests moderate genetic diversity of *D. alata* accessions. Kagera region had the highest mean number of (1.5) private alleles. Analysis of molecular variance revealed that 91% of the variation was attributed to within population while among population contributed 9% of the total variation. The highest Nei's genetic distance (0.65) was for accessions sampled from Arusha and Mtwara regions. Principal coordinate analysis and Unweighted Paired Group Method using Arithmetic (UPGMA) analysis grouped *D. alata* accessions into two major clusters regardless of geographical origin and local names. The Bayesian structure analysis confirmed the two clusters obtained in UPGMA and revealed an admixture of *D. alata* accessions in all six regions suggesting farmers' extensive exchange of planting materials. These results are helpful in the selection of *D. alata* accessions for breeding programs in Tanzania.

Introduction

Yam (*Dioscorea* spp.) is an important food security tuber crop supporting more than 300 million people worldwide [1]. The crop is mainly grown in the tropical and subtropical regions of the world, especially in the yam belt of West African countries [2, 3]. In countries where yam is highly cultivated, it is a staple food due to its high carbohydrate (about 77.5% of dry matter) and other mineral contents [4]. The genus *Dioscorea* contains more than 650 species whereby 10 being cultivated while many others are wild species [5].

Water yam (*Dioscorea alata*), also known as greater or winged yam, is a dioecious and polyploid *Dioscorea* species with a ploidy level (2n= 40 to 80) [6]. The primary chromosome number of *D. alata* is 20 [6, 7]. *D. alata* ranks second in production after Guinea yam [8], although it is the most widely distributed yam globally [9]. *D alata* has high importance in food security than other cultivated yam species due to its high yield potential, tuber storability and ease of propagation due to the production of bulbils [10].

Assessing yam genetic diversity is essential for germplasm management, conservation and planning for the development of new varieties [11]. The level of genetic diversity in *D. alata* can be assessed using various molecular markers. Using Random Amplified Polymorphic DNA (RAPD) and Inter Simple Sequence Repeat (ISSR) markers, [12] reported high variability among *D. alata* genotypes. The genetic diversity of *D. alata* accessions from West and Central Africa and Puerto Rico was assessed using Amplified Fragment Length Polymorphism (AFLP) markers and the accessions clustered into three groups, irrespective of the geographical pattern [13]. Using Single Nucleotide Polymorphism (SNP) markers, [14] established the genetic diversity of 100 *D. alata* accessions and reported three genetic groups. Simple Sequence Repeat (SSR)

markers are also among the most commonly used markers in the diversity analysis of crops, including yams. Despite being a relatively old method, SSR is less expensive than advanced techniques such as Diversity Array Technology (DarT) and Next-generation Sequencing (NGS). SSR markers are also highly preferred due to their reproducibility, transferability among related species and co-dominance nature of inheritance [15]. Using 24 SSR markers, [16] established the genetic diversity and population structure of 384 *D. alata* accessions from the South Pacific, Asia, Africa and the Caribbean and revealed wide genetic diversity in six populations. Another study by [17] estimated the genetic diversity of 26 *D. alata* landraces from China using 9 SSR markers and reported four clusters among yam landraces.

In Tanzania, small-scale farmers mainly produce yam for food, income generation and medicinal purposes. The major yam-producing areas in Tanzania are considered to be Mtwara, Lindi, Morogoro, Arusha, Kilimanjaro, Kagera, Coastal, Zanzibar Islands Mbeya and Ruvuma regions. However, despite the importance of yam to especially farmers, there is limited research on this crop. The genetic diversity of yam accessions grown in the country has never been estimated and documented. Since water yam is the most widely cultivated species in the country, the genetic diversity of 63 *D. alata* accessions from major yam-growing regions was estimated using ten polymorphic SSR markers. This information is crucial to understanding the genetic structure of *D. alata* accessions, which will enhance management, conservation and planning for use in breeding programs in Tanzania.

Materials and Methods

Plant materials

The 63 water yam accessions used in this study. The *D. alata* tubers were collected from farmer's fields in six major growing regions in Tanzania during harvesting season between August and October 2019 (Fig 1) and (Table 1). Tubers were maintained for 2 months to break the dormancy and planted to collect leaves for DNA extraction. Large tubers were sliced into minisett of about 60 grams and planted on ridges at Tanzania Agricultural Research Institute (TARI-Kibaha) experimental plots. Standard cultural agronomic practices, including hand weeding and stacking were employed. Plants were manually irrigated once per day ~~in~~ three times a week. Three young fresh leaves were collected (from the replicates of each accession) two months post planting in a zip bag and transported to the International Institute of Tropical Agriculture - Tanzania for DNA extraction.

Fig 1. Map of Tanzania showing regions and study sites where yam samples were collected.

Table 1. Description of 63 *D. alata* accessions used in this study.

S/n	Accession	Local name	Distinct morphological character	Region
1	YM1	Kiraira	Winged stem	Kagera
2	YM2	Kanyanyinyi	Winged stem	Kagera
3	YM3	Kiraira	Winged stem	Kagera
4	YM4	Kiraira	Winged stem	Kagera
5	YM5	Kanyanyinyi	Winged stem	Kagera
6	YM6	Kiraira	Winged stem	Kagera
7	YM7	Kiraira	Winged stem	Kagera
8	YM8	Kiraira	Winged stem	Kagera
9	YM9	Kanyanyinyi	Winged stem	Kagera
10	YM10	Kanyanyinyi	Winged stem	Kagera
11	YM11	Kanyanyinyi	Winged stem	Kagera
12	YM12	Kanyanyinyi	Winged stem	Kagera
13	YM13	Kanyanyinyi	Winged stem	Kagera
14	YM14	Ifure	Winged stem	Arusha

S/n	Accession	Local name	Distinct morphological character	Region
15	YM15	Ifure	Winged stem	Arusha
16	YM16	Ifure	Winged stem	Kilimanjaro
17	YM17	Ifure	Winged stem	Kilimanjaro
18	YM18	Ifure	Winged stem	Kilimanjaro
19	YM19	Ifure	Winged stem	Kilimanjaro
20	YM20	Kijabo	Winged stem	Lindi
21	YM21	Mapeta	Winged stem	Lindi
22	YM22	Nkwande	Winged stem	Lindi
23	YM23	Mapeta	Winged stem	Lindi
24	YM24	Katuri	Winged stem	Lindi
25	YM25	Vijabo	Winged stem	Lindi
26	YM26	Vijabo	Winged stem	Lindi
27	YM27	Vijabo	Winged stem	Lindi
28	YM28	Mapeta	Winged stem	Lindi
29	YM29	Mikirachi	Winged stem	Lindi
30	YM30	Mapeta	Winged stem	Lindi
31	YM31	Vigonzo	Winged stem	Morogoro
32	YM32	Msagala	Winged stem	Morogoro
33	YM33	Vigonzo	Winged stem	Morogoro
34	YM34	Msagala	Winged stem	Morogoro
35	YM35	Mgendagenda	Winged stem	Morogoro
36	YM36	Mgendagenda	Winged stem	Morogoro
37	YM37	Mgendagenda	Winged stem	Morogoro
38	YM38	Mgendagenda	Winged stem	Morogoro
39	YM39	Mgendagenda	Winged stem	Morogoro
40	YM40	Mnangilangi	Winged stem	Mtwara
41	YM41	Mnangilangi	Winged stem	Mtwara
42	YM42	Vitungula	Winged stem	Mtwara
43	YM43	Mnangilangi	Winged stem	Mtwara
44	YM44	Mnangilangi	Winged stem	Mtwara
45	YM45	Hamandeke	Winged stem	Mtwara
46	YM46	Hamandeke	Winged stem	Mtwara
47	YM47	Hamandeke	Winged stem	Mtwara
48	YM48	Mnangilangi	Winged stem	Mtwara
49	YM49	Hangadi	Winged stem	Mtwara
50	YM50	Mnangilangi	Winged stem	Mtwara
51	YM51	Hangadi	Winged stem	Mtwara
52	YM52	Nyuvele	Winged stem	Mtwara
53	YM53	Nyuvele	Winged stem	Mtwara
54	YM54	Nyuvele	Winged stem	Mtwara
55	YM54	Ihumihumi	Winged stem	Mtwara
56	YM56	Nyuvele	Winged stem	Mtwara
57	YM57	Ihumihumi	Winged stem	Mtwara
58	YM58	Mkonga	Winged stem	Mtwara

S/n	Accession	Local name	Distinct morphological character	Region
59	YM59	Mikirachi	Winged stem	Mtwara
60	YM60	Mkonga	Winged stem	Mtwara
61	YM61	Vitungula	Winged stem	Mtwara
62	YM62	Mapeta	Winged stem	Mtwara
63	YM63	Mapeta	Winged stem	Mtwara

DNA extraction and quantification

Genomic DNA was extracted from 300 mg of fresh leaves following the cetyltrimethylammonium bromide (CTAB) protocol [18]. The quality of DNA was visualized by electrophoresis on a 1% agarose gel. The concentration of DNA was estimated using a spectrophotometer at 260 nm wavelength, and DNA was diluted to obtain a working concentration of 25 ng/µl.

Polymerase chain reaction (PCR) and Fragment analysis

A total of 15 SSR markers were screened using all 63 *D. alata* samples used in this study (Table 2). However, only 10 SSR markers were polymorphic and therefore were included in the analysis. PCR was carried out in a total volume of 15 µl containing 25 ng/µl of genomic DNA, 0.2 µM of forward and reverse primers and 7.5 µL of OneTaq Quick-Load 2X Master Mix with Standard Buffer (New England Biolabs, Massachusetts, USA). The PCR program was as follows; denaturation at 94 ºC for 4 min followed by 35 cycles of 94 ºC for 30 sec, annealing temperature (various as shown in Table 2) and 72 ºC for 1 min. The final extension was held at 72 ºC for 7 min. To prove amplification, 3 µl of the PCR products were run on 1.5 % agarose gel (aMReSCO, Solon, Ohio, USA). The PCR products were then sent to Bioscience eastern and central Africa (BecA) Hub in Nairobi, Kenya and were separated on the ABI-3730 capillary

electrophoresis (Applied Biosystems). Data was captured and the resulting fragments were scored using GeneMapper V6 software.

Table 2. Description of SSR markers used to genotype 63 *D. alata* accessions collected from Tanzania. Markers marked with an asterisk (*) were not used in the analysis.

SN	Marker	Forward primer (5' - 3')	Reverse primer (5' – 3')	Motif	Ta
1	Da1A01	TATAATCGGCCAGAGG	TGTTGGAAGCATAGAGAA	$(GT)_8$	51
2	Dab2C05	CCCATGCTTGTAGTTGT	TGCTCACCTCTTTACTTG	$(GA)_{19}$	51
3	Dab2D08	ACAAGAGAACCGACATAGT	GATTTGCTTTGAGTCCTT	$(AG)_{16}$	51
4	Dab2E07	TTGAACCTTGACTTTGGT	GAGTTCCTGTCCTTGGT	$(CT)_{23}$	51
5	Dpr3D06	ATAGGAAGGCAATCAGG	ACCCATCGTCTTACCC	$(GA)_{15}$	51
6	Dpr3F12	TCCCCATAGAAACAAAGT	TCAAGCAAGAGAAGGTG	$(GA)_{16}$	51
7	Da1F08	AATGCTTCGTAATCCAAC	CTATAAGGAATTGGTGCC	$(TG)_{13}$	51
8	H2	AAACCAAACAGGCAAAGCAT	TGCCCTGCTTGTAAGATTGA	$(CA)_9$	56
9	F1	ATGGCTCAAGAGCACACG	GGGCCTCATAAACATGCAAT	$(TA)_5$	60
10	YM80	CCGCCCAATCACATCACATC	TCCCAAGAAGTCTGAGCCG	$(CTT)_{13}$	60
11	A4*	TTCGTTCTCGATAGCGGACT	CCAGTTCCCAGCCTCTTGT	$(CT)_2(GAA)_3GA(GAA)_3$	60
12	YM13*	CCAATCACATCACGTCTAGTCT	GACAATAGAAACTTCGAGACCC	$(CTT)_8$	60
13	H12*	TTGTAATTGGGTGTTGTATTTGC	CGGCCAAAACATTTTCTGAT	$(AT)_6$	56
14	Dpr3F04*	AGACTCTTGCTCATGT	GCCTTGTTACTTTATTC	$(AG)_{15}$	51
15	Da1D08*	GATGCTATGAACACAACTAA	TTTGACAGTGAGAATGGA	$(CA)_8$	51

Source: [19-21]

Data analysis

Genetic diversity analysis

Genetic diversity parameters including the number of alleles per locus, number of different alleles, number of effective alleles, Shannon's Information Index, observed heterozygosity, gene

diversity, inbreeding coefficient and principal coordinate analysis were determined using GenAlEx software version 6.503 [22]. Polymorphic Information Content (PIC) was estimated using PowerMarker software V3.25 [23].

Analysis of molecular variance and Cluster analysis

To ~~quantify~~ the diversity level and genetic relationship among the *D. alata* accessions, Analysis of Molecular Variance (AMOVA) was estimated using GenAlEx software version 6.503 [22]. The Unweighted Paired Group Method using arithmetic Average (UPGMA) was used to construct the dendrogram in PowerMarker software V3.25 [23]. The dendrogram was then generated in Molecular Evolutionary Genetics Analysis (MEGA-X) V10.1.8 [24].

Structure analysis

Bayesian analysis using Structure software V2.3.4 [25] was used to estimate the population's genetic structure. The admixture model for K values from 1 to 10 with a burn-in period of 10,000 steps and 100,000 interactions MCMC (Markov Chain Monte Carlo) was used in the analysis. The most likely optimal number of K clusters was estimated using the ad hoc parameter (ΔK) method [26] in a Structure Harvester [27].

Results

Characteristics of the SSR markers

The Polymorphic Information Content (PIC) values of the ten SSR markers used ranged from 0.33 for the marker F1 to 0.86 for the marker Da1A01, with a mean of 0.63 (Table 3). The high mean PIC obtained implies that the SSR markers used in our study were very informative with high discriminating power. Hence, these markers can be used in genetic diversity analysis.

Genetic diversity of *D. alata*

Genetic diversity parameters are presented in Table 3. The total number of alleles detected per locus was 76, ranging from 4 to 12, with a mean of 7.60. The lowest numbers of alleles per locus were detected in marker F1 while the highest was detected in markers Dpr3F12 and YM80. The effective number of alleles per locus ranged from 1.17 to 4.01, with a mean of 2.47. The Dab2C05 and Dab2E07 markers had the lowest and highest number of effective allele locus per locus, respectively. Shannon information index ranged from 0.29 to 1.47 with a mean of 0.92. The lowest and highest Shannon information index per locus was detected in markers F1 and Dab2E07, respectively. The expected heterozygosity ranged from 0.17 to 0.74 with a mean of 0.49, whereby the lowest and highest expected heterozygosity was detected in markers F1 and Dab2E07, respectively. The inbreeding coefficient ranged from -0.32 to 0.94, with a mean of 0.24. Markers Dab2E07 and Dpr3D06 had the lowest and highest inbreeding coefficient, respectively. Four markers (40%) showed a negative inbreeding coefficient, indicating an excess of heterozygosity.

Table 3. Summary of genetic parameters of ten SSR markers used in assessing the genetic diversity of 63 *D. alata* accessions.

Locus	No. of Alleles	Ne	I	Ho	He	F_{IS}	PIC
Da1A01	9	3.76	1.39	0.76	0.71	-0.09	0.86
Da1F08	6	3.44	1.28	0.73	0.69	-0.05	0.70
Dab2C05	7	1.17	0.35	0.16	0.19	0.28	0.44
Dab2D08	7	2.66	1.06	0.69	0.58	-0.16	0.71
Dab2E07	8	4.01	1.47	0.97	0.74	-0.32	0.85
Dpr3D06	6	1.55	0.61	0.02	0.38	0.94	0.55
Dpr3F12	12	2.54	1.10	0.41	0.59	0.28	0.69
F1	4	1.26	0.29	0.06	0.17	0.61	0.33
H2	5	1.69	0.64	0.05	0.38	0.87	0.48
YM80	12	2.62	0.94	0.33	0.46	0.30	0.67
Mean	7.6	2.47	0.92	0.42	0.49	0.24	0.63

Ne= Number of effective alleles, I= Shannon's diversity index, Ho= Observed heterozygosity, He= Expected heterozygosity, F_{IS} = Inbreeding coefficient and PIC= Polymorphic Information content.

Genetic diversity of *D. alata* based on population

The genetic diversity among and within 63 *D. alata* accessions based on the population is presented in Table 4. The number of effective loci was lowest (1.78) in Kilimajaro region and highest (3.32) in Kagera region, with a mean of 2.47. Shannon Information Index was lowest (0.60) in the Kilimanjaro region and highest (1.22) in Kagera region, with a mean of 0.92. The mean number of private alleles ranged from 0.10 (Arusha region) to 1.5 (Kagera region), with a mean of 1.03. Observed heterozygosity (Ho) ranged from 0.32 to 0.55 in Mtwara and Kagera regions, respectively, with a mean of 0.42. The expected heterozygosity (He) ranged from 0.35 to 0.60 in Kilimanjaro and Kagera regions, respectively, with a mean of 0.49. Our results indicate that *D. alata* accessions collected from Kagera region had the highest genetic diversity followed by Mtwara region. The mean polymorphic loci were 75.71%.

Table 4. Genetic diversity of 63 *D. alata* accessions based on populations as generated by 10 SSR markers.

Population	Ne	I	Private allele	Ho	He	% Poly loci
Kagera	3.32	1.22	1.50	0.55	0.60	100
Arusha	2.13	0.75	0.10	0.40	0.46	80
Kilimanjaro	1.78	0.60	0.30	0.41	0.35	60
Lindi	2.47	0.95	0.20	0.42	0.50	90
Morogoro	2.33	0.84	0.30	0.41	0.45	90
Mtwara	2.79	1.14	0.70	0.32	0.57	100
Mean	2.47	0.92		0.42	0.49	75.71

Ne= Number of effective alleles, I= Shannon diversity index, Ho= Observed heterozygosity, He= Expected heterozygosity and % Poly Loci= Percentage of polymorphic loci.

Analysis of molecular variance (AMOVA)

Analysis of molecular variance showed a highly significant difference ($p<0.001$) in *D. alata* accessions within regions. The analysis showed that 91% of the variation was within the

population, while only 9% of the total variation was among the population (Table 5). The genetic differentiation index (F_{ST}= 0.09) indicates low genetic differentiation among regions.

Table 5. Analysis of Molecular Variance (AMOVA) of the SSR markers among and within 63 *D. alata* accessions.

Source	DF	SS	MS	Estimated	Percentage	*p*
Among pops	5	45.48	9.09	0.32	9%	0.001
Within pops	120	369.00	3.08	3.07	91%	
Total	125	414.48		3.39	100%	
F- Statistics						
F_{ST}= 0.09						

DF= Degree of freedom, SS= Sum of squares and MS= Mean sum of square, F_{ST}= Genetic differentiation index

Principal coordinate analysis (PCoA)

The first three principal coordinate axes accounted for 41.19% of the total variation (Table 6). The PCoA of the correlation between the genetic relationship of *D. alata* accessions and geographical distribution showed that *D. alata* accessions were divided into two groups (Fig 2). Group A contained *D. alata* accessions collected from all six regions, while group B had accessions from Kagera, Morogoro and Lindi regions. The scatter plot grouped *D. alata* accessions regardless of their geographical origin and local names.

Table 6. Eigenvalue and percentage variation observed at the first 3-axis principal coordinate analysis.

Axis	Eigenvalue	Percentage variation	Cumulative percentage variation
1	13.56	17.89	17.89
2	10.53	13.89	31.78
3	7.14	9.42	41.19

Fig 2. Principal coordinate analysis (PCoA) showing the relationship among 63 *D. alata* accessions using 10 SSR markers. Group A contained accessions collected from all six regions while group B had accessions from Kagera, Lindi and Morogoro. Coord. 1 and Coord. 2 represent the first and second coordinates respectively.

Genetic identity and genetic distance

The Nei's unbiased genetic identity and genetic distances are presented in Table 7. The genetic distance among accessions and regions varied from 0.01 to 0.65. The highest genetic distance was for accessions collected from Arusha and Mtwara (0.65), followed by Kagera and Kilimanjaro (0.58), while the lowest genetic distance was for accessions from Lindi and Morogoro (0.01). Genetic identity between accessions and regions varied from 0.52 to 0.99. The highest genetic identity was for accession from Morogoro and Lindi (0.99), while the lowest was for accessions collected from Mtwara and Arusha (0.52).

Table 7. Nei's Unbiased Genetic identity and genetic distance of 63 *D. alata* accessions using ten SRR markers.

Location	Arusha	Kagera	Kilimanjaro	Lindi	Morogoro	Mtwara
		Genetic identity				
Arusha		0.72	0.54	0.60	0.71	0.52
Kagera	0.33		0.56	0.73	0.72	0.72
Kilimanjaro	0.31	0.58		0.65	0.66	0.57
Lindi	0.51	0.31	0.43		0.99	0.90
Morogoro	0.35	0.32	0.42	0.01		0.83
Mtwara	0.65	0.32	0.57	0.10	0.19	
				Genetic distance		

Cluster analysis

UPGMA grouped the 63 *D. alata* accessions into two major clusters, A and B (Fig 3). Generally, the accessions clustered regardless of their geographical origin. Clusters A and B had 49 and 14 accessions, respectively. Cluster A was further subdivided into two sub-clusters (A1 and A2). Sub-cluster A1 had 28 accessions collected from all regions surveyed, while sub-cluster A2 had 21 accessions collected from Kagera, Morogoro, Lindi and Mtwara regions. Cluster B had two sub-clusters (B1 and B2). Sub-cluster B1 contained 5 accessions from the

Kagera region, while sub-cluster B2 had 9 accessions collected from Morogoro, Lindi and Mtwara regions. In group B1, accessions collected from Kagera region clustered together, suggesting a distant relationship from the rest.

Fig 3. UPGMA cluster analysis of 63 *D. alata* accessions based on 10 SSR markers, using MEGA-X software. A1 and A2 are sub-clusters of cluster A while B1 and B2 represent sub-clusters of cluster B

Structure analysis

The Bayesian analysis performed in STRUCTURE software for the SSR data confirmed the two clusters obtained in the UPGMA. Evanno's method showed a ΔK peak value of K=2, suggesting the presence of two distinct clusters, which confirmed that the *D. alata* accessions collected from the six regions are genetically structured in two groups (Fig 4). The STRUCTURE results are consistent with the UPGMA cluster in that all regions are admixed, and only Kilimanjaro region had a nearly homogeneous population.

Fig 4. Structure of the genetic diversity of 63 *D. alata* accessions based on Bayesian analysis of 10 SSR markers at K=2. The vertical bar represents yam accessions assigned into two clusters. KG= Kagera, AR= Arusha, KL= Kilimanjaro, LD= Lindi, MR= Morogoro and MT= Mtwara region

Discussion

Estimating the genetic diversity of yam is very important in understanding the extent of genetic variation available for proper germplasm management and planning for breeding programs [11, 28].

SSR markers used in our study were highly polymorphic, suggesting that these markers were able to reveal variations within the accessions. PIC is an essential feature in evaluating the informativeness of a molecular marker [29]. Generally, PIC values between 0.5 and 1.0 indicate a high informative marker, whereas values less than 0.5 indicate a narrow informative marker [30]. [31], reported high PIC (0.92) using 11 SSR markers in 89 *D. alata* accessions collected from Brazil. Also, [32] reported high PIC (0.91) in 14 SSR markers used to assess 49 *D. alata* accessions collected from Ghana. On the other hand, [33] reported a lower mean PIC (0.43) using 8 SRR markers in 127 *D. alata* accessions from the International Institute of Tropical Agriculture (IITA) geneBank. Compared to this study, the different PIC levels observed in other studies could be attributed to different SSR markers employed, which target different loci and alos the composition of the yam genotypes.

The low number of alleles per locus observed in this study is an indication of allelic variants per locus (genetic diversity) present in the *D. alata* accessions. A study by [34] reported 97 alleles per locus in 89 *D. alata* accessions collected from nine West African countries, while [32] recorded high (273) alleles per locus in 49 *D. alata* accessions in Ghana. The different number of alleles observed between our study and others, may be due to each using different *D.alata* genotypes and different SSR markers which assess different loci. The composition of yam assessed in our study are mainly *D. alata* accessions collected from farmers fields.

The mean effective number of alleles per locus obtained in our study could indicate moderate genetic diversity of *D. alata* accessions due to spartial dispersion of accessions in the study sites. Different number of effective alleles were reported in previous studies. A study by [17] reported 2.26 effective number of alleles using nine SSR markers in 26 *D. alata* germplasm from Southern China while [35] reported 1.71 effective number of alleles using 10 SSR markers in 33 yam accessions collected from Ethiopia. Generally, the differences in the number of effective alleles can reflect the genetic diversity of the population. The greater the value of the number of effective alleles the higher the genetic diversity of the population [36].

The mean Shannon diversity index observed in this study indicates moderate genetic diversity among *D. alata* accessions. The Shannon diversity value from this study is higher compared to 0.78 reported by [37] in *D. alata* accessions from China and ~~slightly~~ lower than 1.29 observed by [38] in *D. alata* cultivars from Brazil. As a vegetatively propagated crop, yam tends to maintain a high level of heterozygosity [39, 40]. However, due to the presence of few flowers and dioecism, limited genetic recombination may occur in yam [41], which may explain the moderate genetic diversity observed in this study.

The mean expected heterozygosity (the genetic diversity) observed in our study suggests moderate genetic diversity of *D. alata* accessions. The sporadic and narrow distribution of *D. alata* accessions from the study sites could also account for the observed results. The genetic diversity from study is low compared to the 0.66 reported by [16] in *D. alata* accessions collected from South Pacific, Asia, Africa and the Caribbean. Similarly, a study by [32] revealed higher (0.77) genetic diversity in *D. alata* accessions collected from Ghana than the genetic diversity reported in this study. The limited and inconsistence flowering in yam could explain different levels of genetic diversity reported in previous studies compared to our study.

Furthermore, the different levels of genetic diversity could results from geographical patterns of species and life-history [42]. In our study, accessions were collected from regions with sporadic geographical distribution with possibility of both outcrossing and selfing mode of reproduction.

The inbreeding coefficient (F_{IS}) showed that four markers (40%) had a negative inbreeding coefficient, indicating an excess of heterozygosity within the population. This suggests the possible hybridization of these yam accessions. Similar results by [35] stated an excess of heterozygotes in 3 loci out of 10 SSR markers used in yam accession collected from Ethiopia. Similarly, [43] observed that 3 of 7 SSR markers had an excess of the heterozygotes in Guinea yam accessions collected from Ethiopia. The observed excess heterozygosity implies the presence of multiple demes within the population [43]. In our study, the private alleles were higher in Kagera region and lowest in Arusha region. Private alleles indicate the existence of unique genes that have evolutionary significance. *D. alata* accession from the Kagera region showed the highest number of private alleles, higher genetic diversity and higher Shannon diversity index. Therefore, Kagera region is rich in yam diversity compared to the rest of the studied regions.

The AMOVA results in thi study revealed that higher variation is attributed to within the population and low to among population. The low variation observed among the population in our study suggests a high rate of germplasm exchange between neighbor regions. It also indicates that there is no significant geographical differentiation within *D. alata* accessions from the study sites. High variation within the population could be due to recombination, mutation, cross-pollination, or other processes that can produce new genes and alleles [44]. A similar study by [31] revealed higher variation within the population (95.91%), while among the population, was only 4.09%. Similarly, [45] reported that 96% of the variation was within population while 4

% was attributed to among population. The high within population variation observed implies a lack of genetic structure.

The principal coordinate analysis showed the dispersion of *D. alata* accessions into two groups regardless of their geographical origin. Most accessions from different geographical distributions were clustered within the same groups. The clustering pattern suggests that these *D. alata* accessions are similar despite different local names and geographical origins. The clustering observed in our study could be due to the lack of improved yam varieties which causes farmers to grow similar varieties over the years. Similar results were reported by [41]_while assessing the genetic diversity of 142 *D. alata* cultivars from South China. The authors reported only two groups despite collecting *D. alata* cultivars from eight provinces in China. A study by [46] revealed that grouping of accessions with different names into same group could be due to similar accessions that might be known by different names in different regions.

The genetic distance observed in this study was highest for accessions sampled from Arusha and Mtwara [47] reported that genetic distance is related to geographic distance. The high genetic distance observed between Arusha and Mtwara can be due to less possibility of germplasm exchange between the two regions. Mtwara is in the Southern part of Tanzania, while Arusha is in the North, and the distance apart is more than 1,100 km. However, the high genetic distance observed in this study between the two regions may enhance the possibilities of yam improvement through breeding [48].

The UPGMA cluster analysis grouped 63 *D. alata* accessions into two distinct clusters (A and B) and four sub-clusters. Despite different local names, the dendrogram grouped *D. alata* accessions irrespective of their geographical origin, except for sub-cluster B1, which contained *D. alata* accessions from Kagera region. The grouping observed in our study suggests these

accessions are similar. In contrast, grouping in sub-cluster B1 could suggest that these accessions might have some unique genetic characteristics compared to the rest of the groups. Despite yam accessions being collected from six regions, the two groups observed in this study suggest a lack of improved yam varieties since there is no yam breeding program in the country. The exchange of planting materials between farmers over the years also explains the pattern. Similar studies by [31, 35, 49] observed no geographical pattern among yam accessions collected from Brazil, Benin and Ethiopia, respectively.

The structure analysis confirmed the two clusters generated by UPGMA and showed no clear structure of *D. alata* accessions. The analysis revealed a high admixture rate of *D. alata* accessions between the six sampling regions. The high proportion of admixture observed could be due to the high frequency of exchange of yam genetic resources among farmers from neighboring regions [32] and gene flow among *D. alata* accessions [14]. A similar study by [31] reported admixture between *D. alata* accessions collected from Brazil and attributed the presence of admixture accessions to germplasm exchange between different regions.

Conclusion

This study revealed moderate genetic diversity of *D. alata* accessions from six major growing regions of Tanzania. The study showed that *D. alata* accessions are grouped into two clusters despite different geographical ~~origins and local names~~, which suggest their genetic similarities. The lack of a yam breeding program in the country and extensive farmers' exchange of planting materials may have contributed to the moderate genetic diversity. Information obtained from this study is crucial for selecting *D. alata* accessions for breeding programs and conservation strategies.

Acknowledgments

We thank Ms. Magreth Lupembe of Tanzania Agricultural Research Institute and other staff from Tanzania Agricultural Research Institute at Maruku, Selian and Naliendele for their technical support during the field survey. The authors acknowledge the International Institute of Tropical Agriculture (Tanzania), especially the Molecular lab unit, for their support during laboratory work. We also appreciate the assistance of Ms. Lucy Muthui of the International Livestock Research Institute - SegoliP unit and Dr. Inosters Nzuki of Africa Biosystems Limited, Nairobi, Kenya for the SSR data scoring.

References

1. Siadjeu C, Mayland-Quellhorst E, Albach DC. Genetic diversity and population structure of trifoliate yam (*Dioscorea dumetorum* Kunth) in Cameroon revealed by genotyping-by-sequencing (GBS). BMC plant biology. 2018; 18(1): 1-14. doi: 10.1186/s12870-018-1593-x.

2. Girma G, Spillane C, Gedil M. DNA barcoding of the main cultivated yams and selected wild species in the genus *Dioscorea*. Journal of Systematics and Evolution. 2016; 54(3): 228-37. doi: 10.1111/jse.12183.

3. Girma G, Bhattacharjee R, Lopez-Montes A, Gueye B, Ofodile S, Franco J, et al. Re-defining the yam (*Dioscorea* spp.) core collection using morphological traits. Pla Gen Res. 2018; 16(3): 193-200. doi: 10.1017/S1479262117000144.

4. Bhattacharjee R, Nwadili CO, Saski CA, Paterne A, Scheffler BE, Augusto J, et al. An EST-SSR based genetic linkage map and identification of QTLs for anthracnose disease

resistance in water yam (*Dioscorea alata* L.). PloS one. 2018; 13(10): e0197717. doi: 10.1371/journal.pone.0197717.

5. Ignatius NA, Rachid H, Pierre NS, Achiangia PN. Yam (*Dioscorea* spp.) production trends in Cameroon: A review. African Journal of Agricultural Research. 2019; 14(26): 1097-110. doi: 10.5897/AJAR2019.13978.

6. Arnau G, Némorin A, Maledon E, Abraham K. Revision of ploidy status of *Dioscorea alata* L.(Dioscoreaceae) by cytogenetic and microsatellite segregation analysis. Theoretical and Applied Genetics. 2009; 118(7): 1239-49. doi: 10.1007/s00122-009-0977-6.

7. Mignouna H, Mank R, Ellis T, Van Den Bosch N, Asiedu R, Abang M, et al. A genetic linkage map of water yam (*Dioscorea alata* L.) based on AFLP markers and QTL analysis for anthracnose resistance. Theoretical and Applied Genetics. 2002; 105(5): 726-35. doi: 10.1007/s00122-002-0912-6.

8. Cormier F, Lawac F, Maledon E, Gravillon M-C, Nudol E, Mournet P, et al. A reference high-density genetic map of greater yam (*Dioscorea alata* L.). Theoretical and Applied Genetics. 2019; 132(6): 1733-44. doi: 10.1007/s00122-019-03311-6.

9. Baah F, Maziya-Dixon B, Asiedu R, Oduro I, Ellis W. Nutritional and biochemical composition of *D. alata* (*Dioscorea* spp.) tubers. J Food Agric Environ. 2009; 7(2): 373-8.

10. Sartie A, Asiedu R. Segregation of vegetative and reproductive traits associated with tuber yield and quality in water yam (*Dioscorea alata* L.). Afr J Biotech. 2014; 13(28): 2807- 18. doi: 10.5897/AJB2014.13839.

11. Onda Y, Mochida K. Exploring genetic diversity in plants using high-throughput sequencing techniques. Current Genomics. 2016; 17(4): 358-67.

12. Rao GK, Patel N, Patel A, Desai K, Ahlawat T, Kapadia C. Characterization of greater yam (*Dioscorea alata* L.) genotypes by using morphological markers. Inter J Chem Stud. 2019; 7(3): 4961-7.

13. Egesi C, Asiedu R, Ude G, Ogunyemi S, Egunjobi J. AFLP marker diversity in water yam (*Dioscorea alata* L.). Plant Genetic Resources. 2006; 4(3): 181-7. doi: 10.1079/PGR2006121.

14. Agre P, Asibe F, Darkwa K, Edemodu A, Bauchet G, Asiedu R, et al. Phenotypic and molecular assessment of genetic structure and diversity in a panel of winged yam (*Dioscorea alata*) clones and cultivars. Scientific Reports. 2019; 9(1): 1-11. doi: 10.1038/s41598-019-54761-3.

15. Bohra A, Jha R, Pandey G, Patil PG, Saxena RK, Singh IP, et al. New hypervariable SSR markers for diversity analysis, hybrid purity testing and trait mapping in Pigeonpea [*Cajanus cajan* L.) Millspaugh]. Frontiers in plant science. 2017; 8: 377. doi: 10.3389/fpls.2017.00377.

16. Arnau G, Bhattacharjee R, Sheela M, Malapa R, Lebot V, Abraham K, et al. Understanding the genetic diversity and population structure of yam (*Dioscorea alata* L.) using microsatellite markers. PloS one. 2017; 12(3): e0174150. doi: 10.1371/journal.pone.0174150.

17. Chen X, Sun J, Zhu Q, Xiao Y, Zhang H, Huang Y, et al. Characterizing diversity based on phenotypes and molecular marker analyses of purple yam (*Dioscorea alata* L.) germplasm in southern China. Genetic Resources and Crop Evolution. 2022: 1-13. doi: 10.1007/s10722-022-01388-5.

18. Allen G, Flores-Vergara M, Krasynanski S, Kumar S, Thompson W. A modified protocol for rapid DNA isolation from plant tissues using cetyltrimethylammonium bromide. Nature protocols. 2006; 1(5): 2320. doi: 10.1038/nprot.2006.384.

19. Siqueira MV, Marconi TG, Bonatelli ML, Zucchi MI, Veasey EA. New microsatellite loci for water yam (*Dioscorea alata*, Dioscoreaceae) and cross- amplification for other Dioscorea species. American journal of botany. 2011; 98(6): e144-e6. doi: 10.3732/ajb.1000513.

20. Tostain S, Scarcelli N, Brottier P, Marchand JL, Pham JL, Noyer JL. Development of DNA microsatellite markers in tropical yam (*Dioscorea* sp.). Molecular Ecology Notes. 2006; 6(1): 173-5. doi: 10.1111/j.1471-8286.2005.01182.x.

21. Tamiru M, Yamanaka S, Mitsuoka C, Babil P, Takagi H, Lopez-Montes A, et al. Development of genomic simple sequence repeat markers for yam. Crop science. 2015; 55(5): 2191-200. doi: 10.2135/cropsci2014.10.0725.

22. Peakall R, Smouse PE. GENALEX 6.5: genetic analysis in Excel. Population genetic software for teaching and research-an update. Molecular ecology notes. 2012; 6(1): 288-95. doi: 10.1111/j.1471-8286.2005.01155.x.

23. Liu J. POWERMARKER–A powerful software for marker data analysis. North Carolina State University Bioinformatics Research Center, Raleigh, NC. 2002.

24. Kumar S, Stecher G, Li M, Knyaz C, Tamura K. MEGA X: molecular evolutionary genetics analysis across computing platforms. Molecular biology and evolution. 2018; 35(6): 1547. doi: 10.1093/molbev/msy096.

25. Pritchard JK, Stephens M, Donnelly P. Inference of population structure using multilocus genotype data. Genetics. 2000; 155(2): 945-59. doi: 10.1093/genetics/155.2.945.

26. Evanno G, Regnaut S, Goudet J. Detecting the number of clusters of individuals using the software STRUCTURE: a simulation study. Molecular ecology. 2005; 14(8): 2611-20. doi: 10.1111/j.1365-294X.2005.02553.x.

27. Earl DA, VonHoldt BM. STRUCTURE HARVESTER: a website and program for visualizing STRUCTURE output and implementing the Evanno method. Conservation genetics resources. 2012; 4(2): 359-61. doi: 10.1007/s12686-011-9548-7.

28. Ulukan H. The evolution of cultivated plant species: classical plant breeding versus genetic engineering. Plant Systematics and Evolution. 2009; 280(3): 133-42. doi: 10.1007/s00606-008-0118-8.

29. Ni J, Colowit PM, Mackill DJ. Evaluation of genetic diversity in rice subspecies using microsatellite markers. Crop science. 2002; 42(2): 601-7. doi: 10.2135/cropsci2002.6010.

30. Paál D, Kopernick J, Gasper J, Vašíček D, Vašíčková K, Bauerová M, et al. Microsatellite analysis of the Slovak carniolan honey bee (*Apis mellifera* carnica). Journal of Microbiology, Biotechnology and Food Sciences. 2021; 2021: 1517-25.

31. Siqueira MV, Bonatelli ML, Günther T, Gawenda I, Schmid KJ, Pavinato VA, et al. Water yam (*Dioscorea alata* L.) diversity pattern in Brazil: an analysis with SSR and morphological markers. Genetic resources and crop evolution. 2014; 61(3): 611-24. doi: 10.1007/s10722-013-0063-4.

32. Otoo E, Anokye M, Asare P, Tetteh J. Molecular Categorization of Some Water Yam (*Dioscorea alata* L.) Germplasm in Ghana Using Microsatellite (SSR) Markers. Journal of Agricultural Science and Technology. 2015; 7(10). doi: 10.5539/jas.v7n10p225.

33. Girma G, Gedil M, Spillane C. Morphological, SSR and ploidy analysis of water yam (*Dioscorea alata* L.) accessions for utilization of aerial tubers as planting materials. Genetic resources and crop evolution. 2017; 64(2): 291-305. doi: 10.1007/s10722-015-0351-2.

34. Obidiegwu J, Asiedu R, Ene-Obong E, Muoneke C, Kolesnikova-Allen M. Genetic characterization of some water yam (*Dioscorea alata* L.) accessions in West Africa with simple sequence repeats. Journal of Food, Agriculture & Environment. 2009; 7(3&4): 634-8.

35. Mulualem T, Mekbib F, Shimelis H, Gebre E, Amelework B. Genetic diversity of yam (*Dioscorea* spp.) landrace collections from Ethiopia using simple sequence repeat markers. Australian Journal of Crop Science. 2018; 12(8): 1222-30. doi: 10.21475/ajcs.18.12.08.PNE885.

36. Liu Y, Chen Y, Gong Q, Lai J, Du J, Deng X. Paternity assignment in the polyploid Acipenser dabryanus based on a novel microsatellite marker system. Plos one. 2017; 12(9): e0185280. doi: 10.3389/fgene.2022.908367

37. Cao T, Sun J, Shan N, Chen X, Wang P, Zhu Q, et al. Uncovering the genetic diversity of yams (*Dioscorea* spp.) in China by combining phenotypic trait and molecular marker analyses. Ecology and evolution. 2021; 11(15): 9970-86. doi: 10.1002/ece3.7727.

38. Siqueira MV, Dequigiovanni G, Corazon-Guivin MA, Feltran JC, Veasey EA. DNA fingerprinting of water yam (*Dioscorea alata*) cultivars in Brazil based on microsatellite markers. Horticultura Brasileira. 2012; 30: 653-9. doi: 10.1590/S0102-05362012000400015.

39. Elias M, Penet L, Vindry P, McKey D, Panaud O, Robert T. Unmanaged sexual reproduction and the dynamics of genetic diversity of a vegetatively propagated crop plant, cassava (*Manihot esculenta* Crantz), in a traditional farming system. Molecular Ecology. 2001; 10(8): 1895-907. doi: 10.1046/j.0962-1083.2001.01331.x.

40. Bisognin DA. Breeding vegetatively propagated horticultural crops. Crop Breeding and Applied Biotechnology. 2011; 11: 35-43. doi: 10.1590/S1984-70332011000500006.

41. Wu-Wenqiang, Chen C, Zhang Q, Ahmed JZ, Xu Y, Huang X, et al. A comparative assessment of diversity of greater yam (*Dioscorea alata*) in China. Scientia Hort. 2019; 243: 116-24. doi: 10.1016/j.scienta.2018.08.016.

42. Nybom H. Comparison of different nuclear DNA markers for estimating intraspecific genetic diversity in plants. Molecular ecology. 2004; 13(5): 1143-55. doi: 10.1111/j.1365-294X.2004.02141.x.

43. Mengesha WA, Demissew S, Fay M, Smith R, Nordal I, Wilkin P. Genetic diversity and population structure of Guinea yams and their wild relatives in South and South West Ethiopia as revealed by microsatellite markers. Genetic resources and crop evolution. 2013; 60(2): 529-41. doi: 10.1007/s10722-012-9856-0.

44. Mwirigi P, Kahangi E, Nyende A, Mamati E. Morphological variability within the Kenyan yam (*Dioscorea* spp.). Journal of Applied Biosciences. 2009; 16: 894-901.

45. Loko YL, Bhattacharjee R, Agre AP, Dossou-Aminon I, Orobiyi A, Djedatin GL, et al. Genetic diversity and relationship of Guinea yam (*Dioscorea cayenensis* Lam.–*D. rotundata* Poir. complex) germplasm in Benin (West Africa) using microsatellite markers. Genetic Resources and Crop Evolution. 2017; 64(6): 1205-19. doi: 10.1007/s10722-016-0430-z.

46. Asfaw BT, Worojie TB, Mengesha WA. Assessing morphological diversity in Ethiopian yams (*Dioscorea* spp.) and its correspondence with folk taxonomy. Systematics and Biodiversity. 2021; 19(5): 471-87. doi: 10.1080/14772000.2021.1890269.

47. Nei M. Genetic distance between populations. The American Naturalist. 1972; 106(949): 283-92. doi: 10.1086/282771.

48. Bekele A, Bekele E. Genetic Diversity Analysis of Ethiopian Yam (*Dioscorea* spp) from Different Geographic Origin Using Simple Sequence Repeat. Journal of Plant Sciences. 2021; 9(3): 96-106. doi: 10.11648/j.jps.20210903.14.

49. Zannou A, Agbicodo E, Zoundjihékpon J, Struik P, Ahanchédé A, Kossou D, et al. Genetic variability in yam cultivars from the Guinea-Sudan zone of Benin assessed by random amplified polymorphic DNA. African journal of biotechnology. 2009; 8(1).

Figure

Click here to access/download;Figure;Fig1.tif.tif

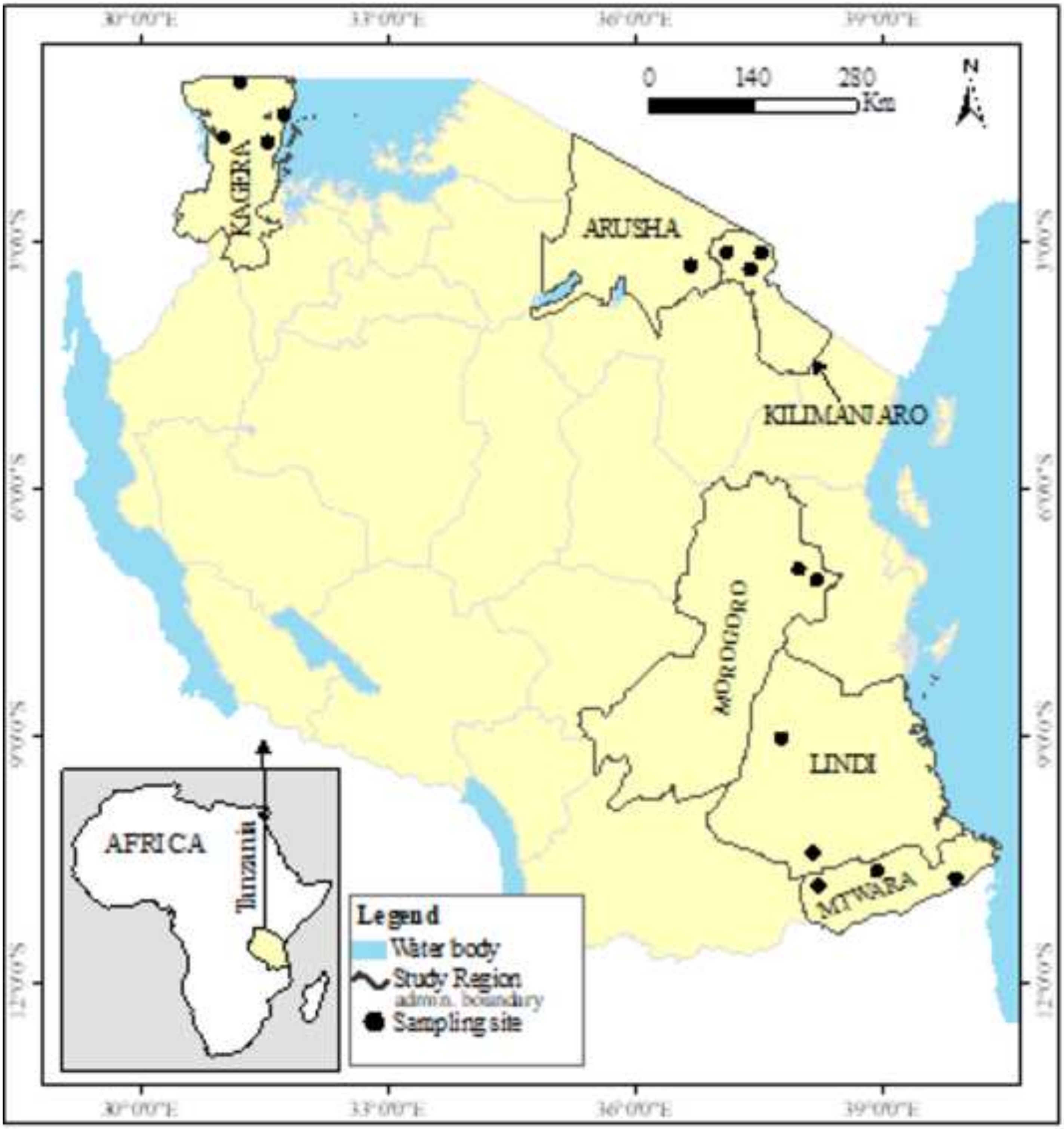

Figure

Click here to access/download;Figure;Fig2.tif.tif

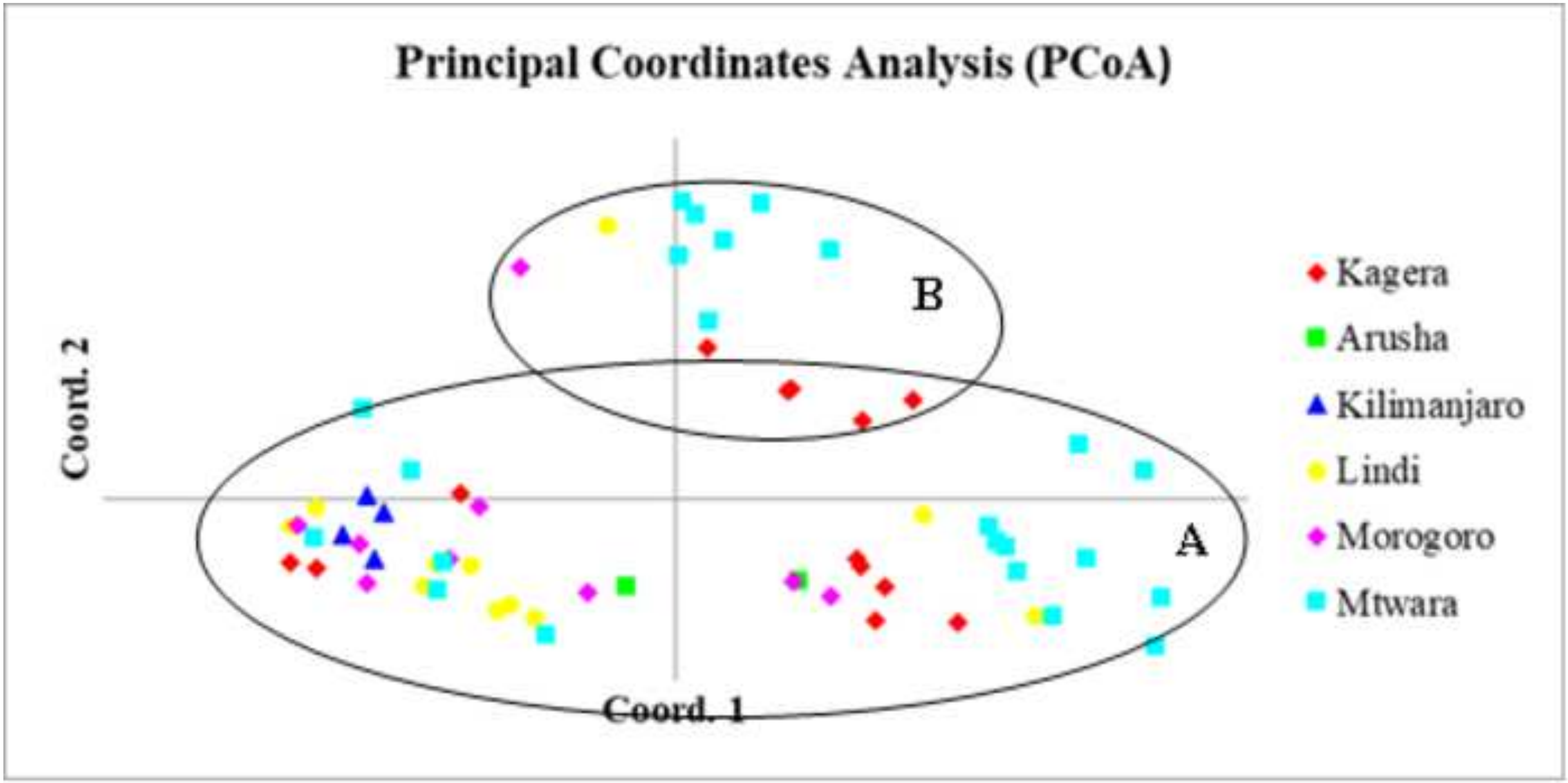

Figure

Click here to access/download;Figure;Fig3.tif.tif

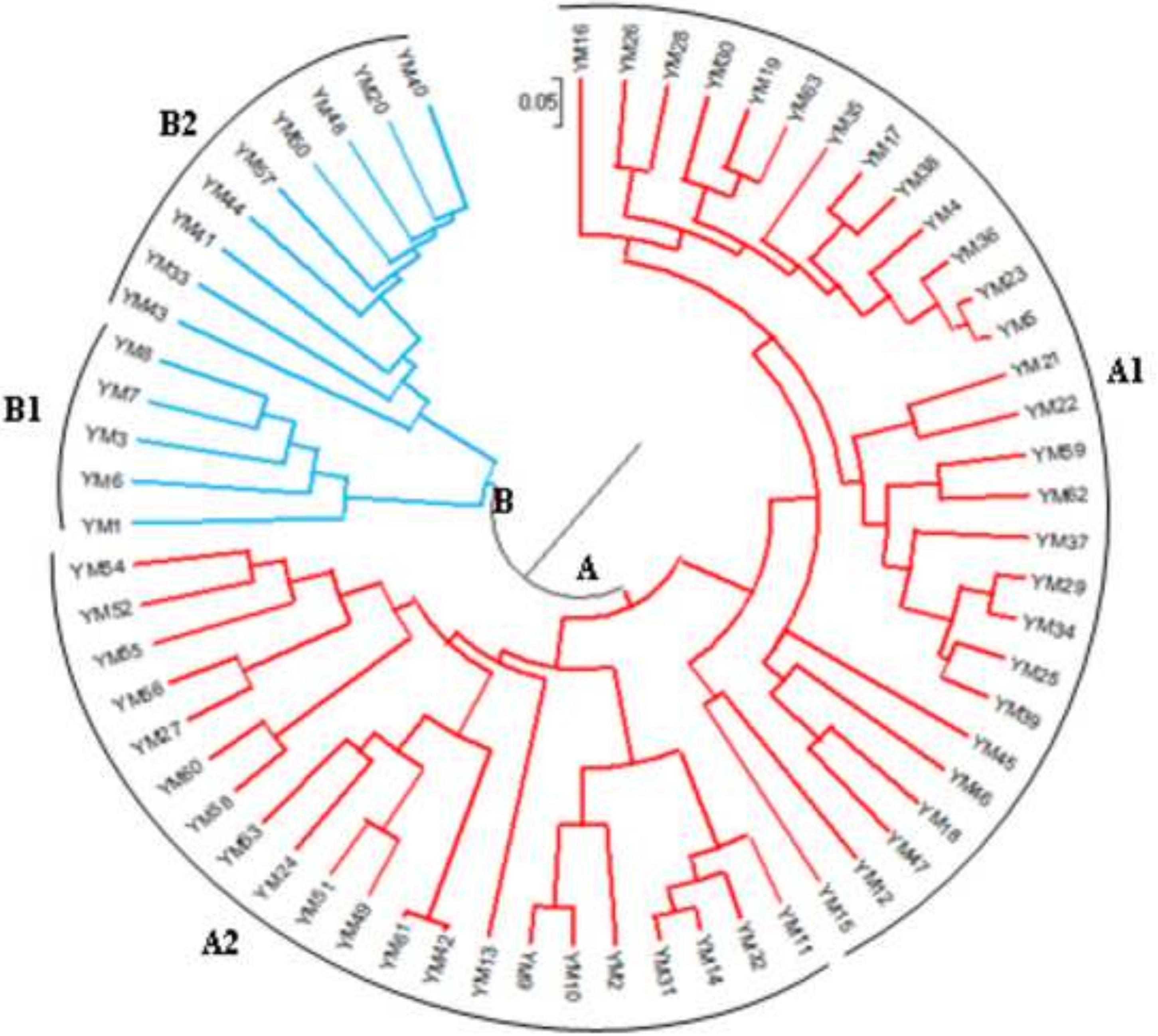

Figure

Click here to access/download;Figure;Fig4.tif.tif

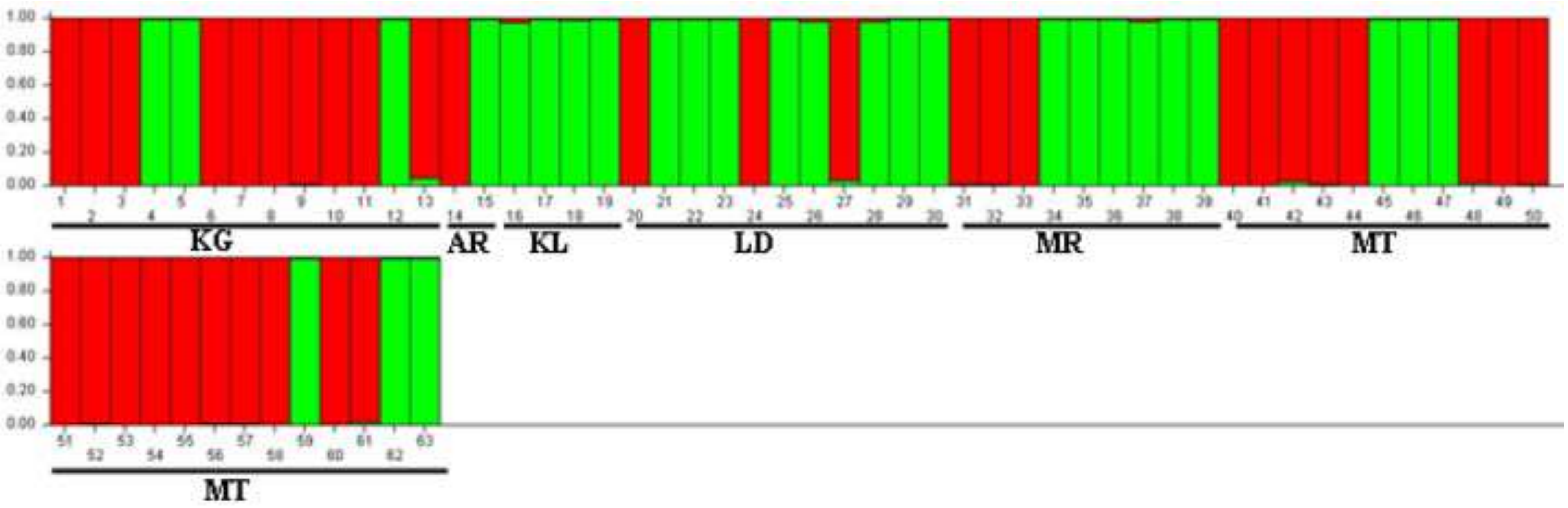

Supplement: Attachment — Submitted filename: PONE-D-22-32759_reviewer with reviewers comment.pdf [file pone.0286480.s001.pdf]
