# Supplementary material for: Unravelling the genetic diversity of water yam (Dioscorea alata L.) accessions from Tanzania using simple sequence repeat (SSR) markers
Source: PLoS One. 2023 May 31;18(5):e0286480. doi: 10.1371/journal.pone.0286480 (PMC10231824; doi:10.1371/journal.pone.0286480)
Supplement: Attachment — Submitted filename: Responses to Reviewers.doc [file pone.0286480.s002.doc]

**PONE-D-22-32759_A rebuttal letter to PLOS ONE**

Dear Chief Editor,

PLOS ONE.

Thank you for the opportunity to submit our revised manuscript with ID # **PONE-D-22-32759** and response to the academic editor and reviewers’ comments. We appreciate the time and expertise you and reviewers dedicated to provide valuable and constructive comments.

We have been able to respond to all suggestions provided by the reviewers. We would like to submit a revised manuscript with track changes and a revised manuscript without track changes.

Here is a matrix with point-by-point response to the academic editor and reviewers’ comments.

I. **Response to academic editor**

| Academic editor comment | Authors response |
| --- | --- |
| Please ensure that your manuscript meets PLOS ONE's style requirements, including those for file naming. | Comment well taken.  All PLOS ONE’s style requirements has been followed. |
| We note that you have stated that you will provide repository information for your data at acceptance. Should your manuscript be accepted for publication, we will hold it until you provide the relevant accession numbers or DOIs necessary to access your data. If you wish to make changes to your Data Availability statement, please describe these changes in your cover letter and we will update your Data Availability statement to reflect the information you provide. | Comment take and addressed.  The dataset has been deposited in Dryad repository (https://doi.org/10.5061/dryad.pg4f4qrv4). Data availability statement has been addressed in the cover letter. |
| Please include your full ethics statement in the ‘Methods’ section of your manuscript file. In your statement, please include the full name of the IRB or ethics committee who approved or waived your study, as well as whether or not you obtained informed written or verbal consent. If consent was waived for your study, please include this information in your statement as well. | Ethics statement has been included in the Material and methods section. |
| We note that Figure 1 in your submission contain map images which may be copyrighted. All PLOS content is published under the Creative Commons Attribution License (CC BY 4.0), which means that the manuscript, images, and Supporting Information files will be freely available online, and any third party is permitted to access, download, copy, distribute, and use these materials in any way, even commercially, with proper attribution. | Thank you for the comment.  Figure 1 was generated using ArcMap 10.3 specifically for this study and it has no any licensing issues. This figure can be used in this study and is permitted to be used freely. Therefore, Figure 1 does not need copyright permission to publish the content specifically under the CC BY 4.0 license. |

I. **Response to reviewers comments**

Reviewer 1

| Reviewer comments | Authors responses |
| --- | --- |
| The present study has been done to estimate the genetic diversity present in the Water yam (Dioscorea alata L.). 63 accessions from six different regions of Tanzania were collected and analyzed using 10 SSR markers. In this study no geographical isolation was found and the accessions shared genetic similarity with each other. The genetic diversity study of Water yam (Dioscorea alata L.) has been done with different research groups using different molecular markers such as RAPD, ISSR, AFLP, SSR and SNP markers. Since this is the first report on genetic diversity of Water yam (Dioscorea alata L.) from Tanzania therefore this study may be interesting for researchers working on this crop. | We appreciate your comments and appreciation to our manuscript. |

**Reviewer 2**

| Reviewer comment | Authors responses |
| --- | --- |
| Line 36 add “cluster analysis” before UPGMA | Comment well taken. The phrase has been added. |
| In Table 1 accession on #54 & 55 are repetitions | Comment well taken. The list of accessions has been rephrased. YM55 has been added. |
| Line 130 what do you mean by number of different alleles? Do you mean number of polymorphic alleles? | Comment well taken. Number of different allele means number of polymorphic alleles. The phrase has been corrected. |
| AMOVA only assess the genetic diversity and genetic relationship among populations not accessions | Comment has been taken. Changes has been incorporated. |
| Why do you use a burn-in –period of 10 000? Some researchers use large burn-in-period. | Thank you for the comment. Burn-in-period of 10 000 has been used in various related studies in structure analysis. However, data has been reanalyzed using a burn-in-period of 100 000. |
| Line 158-160: Shannon information index is a population parameter not locus parameters. It does not has any biological meaning in the case of markers i.e. Shannon information index value for Dab2E07 was 1.47, what does this implies? | Comment take. The sentence and the column with Shannon information index (table 3) has been deleted. |
| Line 173: Some of the regions were not represented by sufficient samples such as Arusha (2) and Kilimanjaro (4), hence the genetic parameter values were under estimated. I suggest to either merging them together or the values should be adjusted based on number of samples per population. | Thank you for your valuable comment. The samples from Arusha and Kilimanjaro has been merged together and represented as samples from Kilimanjaro. Merging these samples is possible because of the close proximity between Arusha and Kilimanjaro regions. The distance from Arumeru (Arusha) to Hai (Kilimanjaro) where accessions were collected is about 40 Km. After merging and analysis, some results in Table 3, Table 4, Table 5 and Table 6 and in-text has changed. |
| Table 5: why among accession variation was not included? GenAlex can give the results even though the percentage variation is 0%. But I doubt that it won't be the case? | Comment well taken. Data has been reanalyzed and the row with data on among individual variation has been included. |
| Figure 2: the classification of the accession is confusing. Based on PCoA 1 & 2 the accessions are classified into three potential groups. If we consider PCoA1, then the A & B classification should be completely different. So please recheck it. | Thank you for your comment. Based on the analysis only two groups (A and B) were found. Within group A there are two sub-groups (A1 and A2). Sub-groups has been included in Figure 2 for clarification. |
| Line 222: It is also ideal to report on the genetic distance between accessions since there may be a chance that the same accession given different name in different regions. | We could not calculate distance between accessions since even the software we used does not provide that information. However, some genetically similar accessions had different local names in different regions. |
| Table 7: The table legend is not informative. Specify the values as genetic distance and identity based on above and below diagonal for ease of reading | Comment well taken. Table 7 legend has been updated to specify the genetic identity and distance. |
| Line 135-136: How is the relationship in terms of number and patterns of clustering between the two clustering approaches i.e. PCA and the dendrogram generated using UPGMA | Comment well taken. The number and pattern of clustering between UPGMA and PCoA were similar. UPGMA clustering confirmed the results obtained by PCoA analysis. The sentence has been added to make this clarification. |
| Figure 4: The quality of Figure 4 should be improve using Excel. | Comment taken and addressed. The quality of the figure has been improved. |
| Citation style when references added at the beginning, in the middle and at the end of the sentence should be rechecked throughout the paper. | Comment well taken. Citation style has been addressed throughout the manuscript. |
| Line 276: Is not clear? Was the low number of alleles due to low or high allelic variants? | Comment well taken. The sentence has been rephrased. |
| Line 276-278: the 97 and 273 values indicated are not the number of alleles per locus, but they are the total number of allele observed overall population. | Comment take. The sentence has been rephrased. |
| Line 303-304: Is flowering time and pattern affected by environmental factors in Yam? If so, explain the results obtained in this study and previous studies based on environmental suitability for flowering. | Comment well taken.  Yes flowering time and pattern in yam is affected by environmental factors. Sentences explaining the same has been added. |
| What do you mean when you say life-history? | Comment well taken. Life-history in this context means annual or perennial nature of the yams. |
| Line 316-318: This might be attributed by the large number of samples collected from Kagera regions unless the values are adjusted based on number of sample per region. This is misleading. | Comment well taken. The number of samples per region has been merged between Kilimanjaro and Arusha. This has improved the reported results. Therefore, the sentence remain unchanged. |
| Line 342-343: The implication is not correct since Arusha is represented by only two accessions. | Comment well taken. The number of samples per region has been merged. Samples from Arusha were merged with samples from Kilimanjaro. The sentence has been rephrased. |
| Line 353-354: The results revealed the lack of wide genetic diversity but not lack of improved variety. The molecular data is all about variation at the DNA sequence level. | Comment taken. The sentence has been rephrased. |

Reviewer 3

| **Reviewer comments** | **Author response** |
| --- | --- |
| In the present investigation, the authors evaluated genetic diversity among 63 accessions of the water yam using 10 SSR markers and reported moderate level of genetic diversity. The genetic diversity in this crop has been widely examined at various locations and using large number of accessions and more advance marker systems such as SNP. The present investigation does not provide any new information’s and the number of accessions and number of markers are very less. Large number of markers evenly distributed across the genome are recommend to be used to assessment of genetic diversity. Therefore, the present study could not be recommended for publication in PLOS One. | Thank you very much for the time devoted in reviewing our manuscript and your comment. Currently, SSR markers are still markers of choice in genetic diversity studies of different crops. However, given the availability of funding, more advanced markers will be employed. On the other hand, this study provided information about the genetic diversity of *D. alata* accessions that are grown in Tanzania. This information is currently lacking in Tanzania, however, the information is very important in breeding and conservation strategies. |
