# Supplementary material for: Unravelling the genetic diversity of water yam (Dioscorea alata L.) accessions from Tanzania using simple sequence repeat (SSR) markers
Source: PLoS One. 2023 May 31;18(5):e0286480. doi: 10.1371/journal.pone.0286480 (PMC10231824; doi:10.1371/journal.pone.0286480)
Supplement: Attachment — Submitted filename: Response to Reviewers.doc [file pone.0286480.s003.doc]

**PONE-D-22-32759_A rebuttal letter to PLOS ONE**

Dear Chief Editor,

PLOS ONE.

Thank you for the opportunity to submit our revised manuscript with ID # **PONE-D-22-32759** and response to the academic editor and reviewers’ comments. We appreciate the time and expertise you and reviewers dedicated to provide valuable and constructive comments.

We have been able to respond to all suggestions provided by the reviewers. We would like to submit a revised manuscript with track changes and a revised manuscript without track changes.

Here is a matrix with point-by-point response to the academic editor and reviewers’ comments.

I. **Journal requirements**

| Academic editor comment | Authors response |
| --- | --- |
| Please review your reference list to ensure that it is complete and correct. If you have cited papers that have been retracted, please include the rationale for doing so in the manuscript text, or remove these references and replace them with relevant current references. Any changes to the reference list should be mentioned in the rebuttal letter that accompanies your revised manuscript. If you need to cite a retracted article, indicate the article’s retracted status in the References list and also include a citation and full reference for the retraction notice. | Comment well taken.  Reference list has been thoroughly checked, the list is complete and correct. No retracted paper has been cited in this manuscript. |

I. **Response to reviewers comments**

Reviewer 1

| Reviewer comments | Authors responses |
| --- | --- |
| The reply to reviewer’s comments point-wise has not been submitted by authors. This needs to be submitted. The corrections in the manuscript needs to be done in track change mode which is missing. Therefore, authors are requested to submit the revised manuscript in the track change mode for further evaluation. | Thank you for the comment. Point-wise reviewer’s comments were submitted in matrix form during previous revision. Further, the revised manuscript with track changes was also submitted in the previous revision. Both attachments are still available in the Editorial manager. |

**Reviewer 2**

| **Reviewer comments** | **Author response** |
| --- | --- |
| The abstract needs to be revisited. They have changed the analysis and the results were different from the original analysis, for example, the AMOVA results have been changed as among population, among individual and within individual. I have also seen few editorial issues to be addressed. | Comment well taken.  The abstract has been updated accordingly. AMOVA and other parameters have been corrected. |
